# Performance evaluation of a novel reticulocyte identification method that uses metachromatic nucleic acid staining based on a crossover analysis of emission DNA/RNA light (RNP Determination™) in hematology analyzer Celltac G+

**DOI:** 10.1111/ijlh.13947

**Published:** 2022-08-17

**Authors:** Kaori Yahagi, Tomoko Arai, Hisako Katagiri, Yoko Yatabe, Hiromitsu Yokota, Yutaka Nagai, Takayuki Mitsuhashi, Masatoshi Wakui, Mitsuru Murata

**Affiliations:** ^1^ Clinical Laboratory Keio University Hospital Tokyo Japan; ^2^ Department of Laboratory Medicine Keio University School of Medicine Tokyo Japan; ^3^ Department of Clinical Laboratory Science Kansai University of Health Sciences Osaka Japan

**Keywords:** Celltac G+, hematology analyzer, performance evaluation, reticulocyte identification, RNP determination

## Abstract

**Introduction:**

Assessing the percentage of reticulocytes (%Retic) is useful for diagnosing and treating blood diseases that present with anaemia. The Celltac G+™ hematology analyzer (HA) uses a novel reticulocyte identification method that involves metachromatic nucleic acid staining with acridine orange and crossover analysis of emission light of DNA/RNA (determination of red cells, nucleic acid‐containing cells, and platelets, RNP Determination™). The red and green fluorescence generated by stained single‐stranded RNA and double‐stranded DNA express immaturity and morphological abnormality of erythrocytes by detecting erythrocyte RNA and DNA content.

**Methods:**

The basic performance of the test automated analyzer (TAA) Celltac G+ was evaluated and compared with the flow cytometry reference method and the comparative automated analyzer (CAA) XN‐1000/2000™. In addition, its precision, limit of quantity (LoQ), linearity, analytical measurement interval (AMI), accuracy, and comparability and the effects of interfering substances were evaluated.

**Results:**

Evaluation of %Retic by the TAA demonstrated good precision and linearity. The AMI was confirmed from 0.02 to 8.23, and the LoQ in %Retic as the coefficient of variation within an 11% limit (*SD*, within a 0.01 limit) was 0.14. TAA correlated well with the reference method and routine HA (CAA). Some deviations were found between TAA and CAA in DNA measurements of erythrocytes from abnormal samples.

**Conclusion:**

Celltac G+ uses a novel measurement principle and can assess erythrocyte immaturity independent of DNA contents. It represents a new HA that provides novel, useful information on immaturity and morphological abnormality of erythrocytes.

## 1 INTRODUCTION

Reticulocytes are de‐nucleated erythrocytes with residual immature RNA and are important for evaluating erythropoiesis in bone marrow. To evaluate the percentage of reticulocytes (%Retic), currently available automated hematology analyzers (HAs) use nucleic acid staining to detect RNA in reticulocytes and differentiate them from mature erythrocytes.^1^ However, these conventional nucleic acid staining methods use a single colour to stain RNA and DNA, which may affect detection of reticulocytes if they contain also DNA. Measurements may be affected also by Interfering substances, which are potentially related to Howell‐Jolly (H‐J) bodies, nucleated erythrocytes, or leukocytes. In addition, interfering mediators, which are assumed to be related to platelets stained brightly by single‐coloured nucleic acid dye, can occur, also in samples from healthy donors. In conventional single‐colour staining, simultaneous passage through laser irradiation of mature erythrocytes without cellular single‐stranded RNA and platelets with staining, for example, of double‐stranded mitochondrial DNA, can cause spurious reticulocyte measurements. Theoretically, errors due to these spurious events may have more impact on measurements when the %Retic is especially low and may have a greater effect in HAs equipped with a flow cytometry measurement (FCM) system. Accurate measurements of the %Retic in the lower range are clinically important for monitoring the early phase of hematopoietic recovery after hematopoietic stem cell transplants. In addition, a marked reduction in reticulocytes (e.g., to less than 0.01 × 10^12^/L) may be found also in acute normocytic anaemia due to acute infection with parvovirus B19. The minimum detection sensitivity of %Retic required to verify such a decrease in reticulocytes is considered to be approximately 0.2.^2^ Therefore, the accuracy of measurement systems, including the minimum detection sensitivity in the low %Retic range, needs to be evaluated.

The prototype Celltac G+™ HA uses a novel reticulocyte identification method that involves metachromatic nucleic acid staining with acridine orange (AO) and is based on a crossover analysis of the light emitted by DNA and RNA^3^ (determination of red cells, nucleic acid‐containing cells, and platelets; RNP Determination™). The red and green fluorescence generated by staining of single‐stranded RNA and double‐stranded DNA reflects immaturity and morphological abnormality of erythrocytes by detecting their RNA and DNA content. We evaluated the performance of RNP Determination™ (RNP) incorporated into Celltac G+. In addition, we investigated the effects of some interfering substances in abnormal samples by using flagging messages obtained from a comparative automated analyzer (CAA).

## 2 MATERIALS AND METHODS

This evaluation was performed according to guidance on the assessment of HAs, including the International Council for Standardization in Haematology (ICSH) recommendations^4^, ^5^ and the Clinical and Laboratory Standards Institute (CLSI) standardized procedure.^5^ All HA and FCM measurements were conducted within 8 h of blood sample collection and completed while the prepared samples were stable.

The study was approved by the local institutional review board (IRB No.: 20180132). Informed consent was obtained from nonpatients by completion of a written informed consent form and from patients by an opt‐out procedure.

### 2.1 Specimen collections

The present study was conducted with 812 peripheral venous blood samples from in‐ and outpatients at Keio University Hospital (Tokyo, Japan). All samples were collected in tubes containing K2‐EDTA (Neotube, 2 ml, OP‐EK0205‐28B[G], NIPRO, Japan).^6^ The blood collection tubes,^7^ blood collection procedure,^8^ and stirring procedure^9^ were all performed according to the methods described by ICSH and CLSI. Analyses were performed on residual samples obtained after routine clinical testing had been completed.

### 2.2 Methods for measuring reticulocytes

#### 2.2.1 Flow cytometry reference method

The flow cytometry method, performed in compliance with the international harmonization protocol CLSI H44‐A2^10^ for %Retic measurement, was used as the reference method. The FACSCantoII™/FACSVia™ (BD Bioscience) instrument was used as the reference flow cytometer, and the following reagents were used according to the standard operating procedures (SOPs) for analysing fresh blood distributed by the external quality assessment survey of the Japanese Society for Laboratory Hematology (JSLH Survey)^11^: Retic‐COUNT™ (FITC: thiazole orange nucleic acid staining reagent)^12^ and immunostaining reagents (2016 panel,^13^ PE: anti‐CD235a positive selection antibody; 2019 panel,^11^ APC: anti‐CD45/CD61/CD41a negative selection antibody). After completion of their review, which was based on the International Harmonization Protocol, the JSLH distributed both SOPs to manufacturers of reference HAs and reference laboratories performing FCMs.

During sample preparation, the negative control samples and thiazole orange single colour nucleic acid‐stained samples (TO‐stained samples) of each specimen were incubated with the immunostaining reagent for 15 min at room temperature (RT) in the dark. After reaction, samples were diluted 200 times: The nucleic acid TO staining reagent was added to the TO‐stained sample (TO‐positive), and 0.1% albumin‐containing phosphate buffer was added to the negative control samples (TO‐negative). Then, samples were incubated for 30 min at RT in the dark. Paired samples (TO‐positive and TO‐negative) were measured, and 5000 events were captured. Each paired sample was analysed by comparing the TO‐positive and TO‐negative plot. A gating strategy was used to determine erythrocyte candidate events by the forward scatter and side scatter profile and the positive‐/negative‐selection antibody and side scatter profile. Erythrocytes were determined in both profiles by AND events in the two erythrocyte candidate events, and the reticulocyte gate was determined from the distribution of unstained sample events. Reticulocytes were calculated from the TO‐stained sample events within the reticulocyte gate. The reference values were calculated from the difference between the stained and unstained samples in the paired reticulocyte gate event, and the mean of the duplicate measurements was used.

#### 2.2.2 CAA XN‐1000/2000

Reticulocytes were assessed also with the CAA XN‐1000/2000™ (Sysmex, Kobe, Japan). This instrument uses the nucleic acid staining reagent Fluorocell RET™ and a two‐dimensional plot (fluorescence intensity and forward‐scattered light intensity).

#### 2.2.3 Test automated analyzer Celltac G+

**FIGURE 1:**
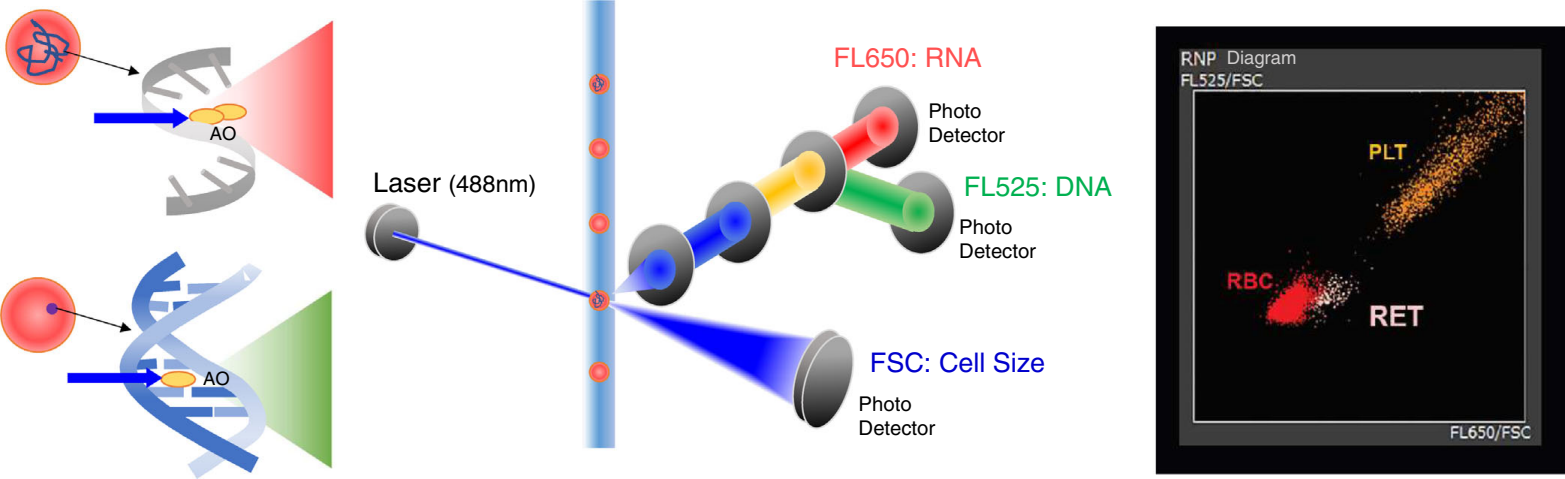
Novel reticulocyte identification method based on RNP Determination™ by metachromatic nucleic acid staining in the haematology analyser Celltac G + .RNP Determination™ is the determination of red cells, nucleic acid‐containing cells, and platelets, and an RNP diagram is a two‐dimensional plot of fluorescence density calculated from information on cell size and fluorescence intensity of two colours, that is, red and green.The RNP diagram allows accurate differentiation between red cells, platelets, and white cells without the use of antibodies. Acridine orange (AO) is a metachromatic cell‐permeant nucleic acid‐binding dye. When irradiated with a blue laser, it emits green fluorescence (525 nm) when bound to double‐stranded DNA and red fluorescence (650 nm) when bound to single‐stranded DNA or RNA. The forward scatter intensity (FSC) obtained by irradiation with a blue laser serves as a cell size marker. When incorporated into a double‐stranded nucleic acid, AO has a fluorescence intensity of 525 nm (FL525); it is first electrostatically bound to phosphoric acid and incorporated into the double strand at a ratio of 1–3 base pairs. At this time, the inserted AO molecules exist as monomers and are not adjacent to each other (intercalation). FL525 is a nucleated cell marker. AO bound to single‐stain DNA or RNA has a fluorescence intensity of 650 nm (FL650); the bound AO molecules exist as dimers by stacking or aggregation. FL650 is as an immaturity cell marker. FL525/FSC expresses the light–dark concentration of green fluorescence in blood cells observed with a fluorescence microscope and the fluorescence density by fluorescence detection of flow cytometry. The FL525/FSC axis represents the amount of double‐stranded DNA divided by blood cell size. FL650/FSC expresses the light–dark concentration of red fluorescence in blood cells observed with a fluorescence microscope and the fluorescence density by fluorescence detection of flow cytometry. The FL650/FSC axis represents the amount of single‐stranded DNA or RNA divided by blood cell size.AO, acridine orange; FL525, fluorescence intensity at 525 nm; FL650, fluorescence intensity at 650 nm; FSC, forward scatter intensity; PLT, platelet; RBC, red cells; RET, reticulocyte.

Celltac G+ with the AO nucleic acid staining reagent MR‐110W™ with an RNP (red and green fluorescence intensities/densities and forward scattered light intensity) was used as the test automated analyzer (TAA) for measuring reticulocytes with a manual loader (ML) and autoloader.^14^ The AO nucleic acid staining reagent enters double‐stranded DNA in the cell nucleus (intercalation) to generate green fluorescence and forms dimer bonds (by stacking or aggregation) with single‐stranded RNA in cells to generate red fluorescence. In the intercalation process, AO is first electrostatically bound to phosphoric acid and then enters the double‐stranded DNA structure at a ratio of 1–3 base pairs. This measurement approach thus differentiates between RNA and DNA (Figure 1),^2^, ^15^, ^16^, ^17^, ^18^ enabling identification of erythrocytes without nuclei and mitochondria, that is, reticulocytes. In comparison, conventional nucleic acid staining is monochromatic, so the fluorescence wavelength cannot identify reticulocytes. In RNP, the fluorescence density of each colour is calculated by dividing the red and green fluorescence intensities by the forward‐scattered light intensity which is cell size information, and the results are plotted in a two‐dimensional manner to create an RNP diagram. The RNP diagram distinguishes between erythrocytes, platelets, and leukocytes without using antibodies. This method has a potential measurement principle to minimize the effect of interfering substances and spurious reticulocyte events and consequently measures reticulocytes more accurately.

### 2.3 Evaluation of basic performance

To evaluate the potential use of the TAA as a routine HA in clinical laboratories, the basic performance of the TAA was evaluated in terms of its precision, limit of quantity (LoQ), linearity, analytical measurement interval (AMI), accuracy, and comparability and the effects of interfering substances. We evaluated both the within‐run precision (repeatability, 10 consecutive measurements)^4^ and the between‐day precision (reproducibility, 10 levels of TAA control materials MK‐RE1/2/3™; twice a day in duplicate measurements). The percentage coefficient of variation (%CV) and *SD* were calculated for repeatability and reproducibility. LoQ was determined from regression analysis of the precision profile for six concentrations in clinical samples^19^ in the preliminary evaluation, which focused on the low concentration range. The linearity, AMI, and accuracy of the TAA and CAA were confirmed by regression analysis of all samples (All) compared with reference FCM, and correlation criteria were validated. The effects of interfering substances and the comparability of the TAA measurements were verified by comparing TAA and CAA values, and correlation criteria were validated. We investigated whether potential interfering substances had any effect on the %Retic measurement by using information obtained from the CAA, such as nucleated red blood cell count (NRBC), white blood cell count (WBC), platelet count (PLT), and flagging messages. FCM results were used as grouping criteria to evaluate accuracy, and the mean values of CAA and TAA were used to evaluate comparability.

### 2.4 Preliminary evaluation

We performed a preliminary evaluation by focusing on the low concentration range. Celltac G+ ML was used as the TAA, XN‐1000, was used as the CAA, and the positive selection method with FACSCantoII was performed as the reference FCM. To investigate repeatability, we used six concentrations of reticulocytes (%Retic: 0.1, 0.2, 1.5, 1.9, 3.1 and 4.5). To evaluate linearity and accuracy, we used 14 samples, and to evaluate comparability and effects of interfering substances, 109 samples. LoQ was calculated from the repeatability data within the assessed accuracy range. The difference of the required minimum detection sensitivity for %Retic was compared with the FCM and confirmed in the samples with a %Retic of less than 0.1 (two clinical samples, one processed sample). The TAA and FCM were compared by dividing samples into negative and positive groups on the basis of our clinical laboratory criteria for CAA measurements in positive samples (%Retic of 2.1 or higher, %NRBC of 0.6 or higher, and %FRCs of 3.0 or higher).

### 2.5 Comprehensive evaluation

For the comprehensive evaluation, Celltac G+ was used as the TAA, XN‐2000 was used as the CAA, and the negative selection method with FACSVia was performed as the improved reference method. For repeatability, we used three concentrations (%Retic: 0.4, 3.0, and 10.1). For linearity, AMI, and accuracy, we used 63 samples, and for comparability and effects of interfering substances, 483 samples. For the evaluations of comparability and the effects of interfering substances, the samples were divided into three groups on the basis of the adult %Retic reference interval (0.8–2.2)^1^: low, 0.0–0.7; normal, 0.8–2.2; and high, over 2.3. For samples assessed in the CAA, we compared high concentration samples (%NRBC, PLT, WBC) and flagging (Interpretive program [IP]) messages. To compare morphology assessments, we selected typical cases from another 220 samples, that is, samples with large deviations between TAA and CAA measurements and with potential interfering substances (erythroblasts, H‐J bodies, fragmented erythrocytes, and large platelet).

### 2.6 Statistical analysis

Statistical analyses were performed with Excel® 2010 (Microsoft), MedCalc® 12.7.8.0 (MedCalc Software, Belgium) and Method Validation version 5.10.9 (Analyse‐it Software, United Kingdom). Significant proportional differences and constant differences were validated by Passing‐Bablok regression analysis. The statistical correlation criteria^5^ were as follows: If the 95% confidence interval (95% CI) of the slope included the value 1, a significant proportional difference was considered to exist between the two methods, but if the 95% CI of the Y‐intercept included the value 0, no constant difference was considered to exist. Data were excluded in case of the following: failure to adhere to the study‐specific procedures; instrument, operator‐related, or sample‐related failure; or a data‐invalidating flag, as described in the operating instructions of each instrument.^5^

## 3 RESULTS

### 3.1 Preliminary evaluation

Appendix A1 shows the repeatability of %Retic (mean, 0.10–4.49; *SD* 0.01–0.09; %CV, 1.6–12.1). All three levels of controls were processed over 15 days to assess reproducibility, and the results of %Retic did not exceed the mean/%CV set by the manufacturer (MK‐RE1™, 1.28/8.1; MK‐RE2™, 4.31/3.6; MK‐RE3™, 9.20/2.4).

Table 1 shows the accuracy results for the TAA and CAA. The correlation coefficient (r) of the FCM was 0.99 for the TAA and 0.98 for the CAA. The slopes of the TAA and CAA and the intercept of the TAA satisfied correlation criteria. However, the intercept of the CAA showed a slight positive shift. Linearity was confirmed for both the TAA and CAA by regression analysis of measurements from patient samples, and AMI was confirmed from 0.02 to 5.69. The mean (individual values) of %Retic in the three samples with a minimum detection sensitivity of less than 0.1 were as follows: FCM, 0.03 (0.02, 0.03*, 0.04); TAA, 0.05 (0.06, 0.03*, 0.06); and CAA, 0.15 (0.10, 0.09*, 0.18). The low %Retic values in the processed sample (*) were comparable to those of the two clinical samples. As can be seen in the AMI precision profile in Appendix A2, the LoQ in %Retic as a %CV within an 11 limit (*SD* within a 0.01 limit) was 0.14.

**TABLE 1:** Evaluation of accuracy and comparability of percentage reticulocyte count in the preliminary evaluation.

												Passing–Bablokfit			
			Reticulocyte percentage range								correlation (r)	Intercept		Slope	
Evaluation	Group	n	x	Min	Mean	Max	y	Min	Mean	Max			95% CI		95% CI
Accuracy	All	14	FCM	0.02	1.42	5.69	TAA	0.03	1.60	6.42	**0.99**	**0.1**	0.0–0.3	**1.07**	0.84–1.44
							CAA	0.12	1.55	5.62	**0.98**	**0.2**	0.1–0.2	**0.94**	0.87–1.00
Comparability	All	109	CAA	0.12	2.55	11.0	TAA	0.03	2.47	14.16	**0.97**	**−0.1**	−0.2–−0.0	**1.02**	0.97–1.07
Comparability	Negative	58	CAA	0.06	1.16	1.89	TAA	0.12	1.07	1.92	**0.95**	**0.1**	−0.1–0.1	**1.05**	0.96–1.16
Comparability	Positive	51	CAA	0.12	4.14	11.0	TAA	0.03	4.17	14.16	**0.96**	**−0.2**	−0.5–−0.0	**1.07**	1.00–1.16

*Note*: The groups in the clinical samples for regression analysis were defined as all, negative (without positive samples), and positive (%Retic ≥2.1 or %NRBC ≥0.6 or %FRC ≥3.0 compared with measurements obtained with the comparative automated analyser). CAA, comparative automated analyser XN‐1000; FCM, flow cytometric reference method for reticulocyte count obtained by flow cytometer FACSCantoII; %FRC, percentage of fragmented red cells; %NRBC, percentage of nucleated red cells; %Retic, percentage of reticulocytes; TAA, test automated haematology analyser Celltac G+; 95% CI, 95% confidence interval.

Abbreviations: CAA, comparative automated analyser; CI, confidence interval; FCM, flow cytometry measurement; TAA, test automated analyser.

Table 1 shows the comparability results for the TAA and CAA, the correlation coefficient (r) of TAA was 0.97 overall (*n* = 109), 0.92 for the negative group (*n* = 58), and 0.96 for the positive group (*n* = 51). The breakdown of the positive sample numbers was as follows: %Retic 41; %NRBC, 26; and %FRC, 19. The slopes and the intercept satisfied the correlation criteria in the overall, negative, and positive groups. The difference between the mean %Retic measured with the TAA and CAA was −0.3 to +0.4 in the negative group and −3 to +3 in the positive group. In the positive group, five specimens had a difference of more than ±1.5, one in the positive direction and four in the negative direction.

### 3.2 Comprehensive evaluation

Appendix A1 shows the repeatability of %Retic (mean, 0.39–10.09‐; *SD*, 0.04–0.14; %CV, 1.4–10.6). All three levels of controls were processed over 5 days to account for reproducibility, and none of the %Retic results exceeded the mean/CV set by the manufacturer (MK‐RE1™, 1.74/4.8%; MK‐RE2™, 4.80/3.6%; and MK‐RE3™, 9.7/2.3%).

**FIGURE 2:**
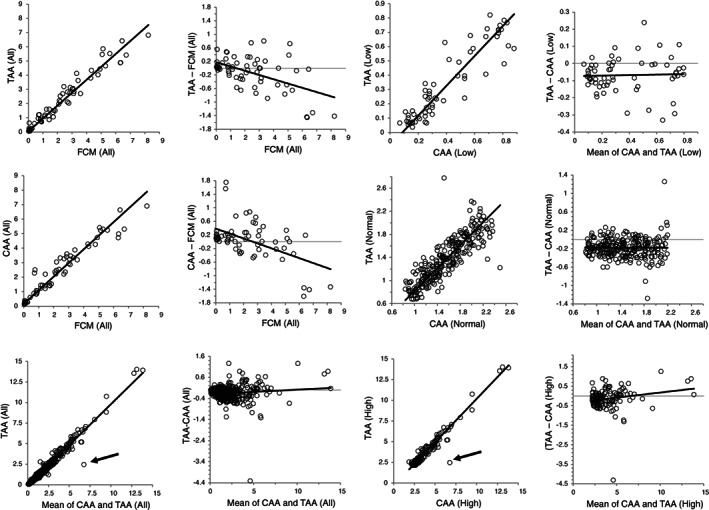
Accuracy and comparative evaluation of the percentage reticulocyte count in the comprehensive evaluation CAA, comparative automated analyser XN‐2000; FCM, flow cytometry reference method for reticulocyte count obtained by flow cytometer, FACSVia; TAA, test automated analyser Celltac G+.

Figure 2 and Table 2 shows the accuracy results for the TAA and CAA. The correlation coefficient (r) of TAA was 0.97 overall, 0.92 for the low group, 0.88 for the normal group, and 0.91 for the high group. The regression equations of the overall samples were as follows: TAA, 0.91 × FCM + 0.0; CAA, 0.94 × FCM + 0.2. For the TAA and CAA, linearity was confirmed by regression analysis in patient samples, and AMI was confirmed from 0.03 to 8.23. The 95% CIs of the TAA and CAA slopes were 0.84–0.99 and 0.87–1.00 overall, 0.98–1.93 and 0.95–3.04 for the low group, 0.86–1.45 and 0.69–1.60 for the normal group, and 0.66–1.06 and 0.62–1.03 for the high group, respectively. The accuracy of %Retic overall and in the low and normal groups was not markedly different between TAA and CAA, but in the high group both instruments showed negative systematic errors versus FCM.

**TABLE 2:** Accuracy and comparability evaluation of the percentage of reticulocytes in the comprehensive evaluation.

												Passing–Bablokfit				Bland–Altman fit					
			Reticulocyte percentage range								correlation (r)	Intercept		Slope		Constant		Intercept		Slope	
Evaluation	Group	n	x	Min	Mean	Max	y	Min	Mean	Max			95% CI		95% CI		95% CI		95% CI		95% CI
Accuracy	All	63	**FCM**	0.0	2.3	8.2	**TAA**	0.1	2.2	6.8	**0.97**	**0.0**	0.0–0.2	**0.91**	0.84–0.99	**−0.1**	−0.2–0.0	**0.2**	0.0–0.3	**−0.1**	−0.2–−0.1
							**CAA**	0.1	2.4	6.9	**0.97**	**0.2**	0.1–0.2	**0.94**	0.87–1.00	**0.0**	−0.1–0.2	**0.4**	0.2–0.6	**−0.1**	−0.2–−0.1
Accuracy	Low	17	**FCM**	0.0	0.2	0.8	**TAA**	0.1	0.4	1.2	**0.92**	**0.0**	−0.1–0.0	**1.57**	0.98–1.93	**0.1**	0.0–0.2	**0.0**	−0.1–0.1	**0.5**	0.1–0.8
							**CAA**	0.1	0.6	2.5	**0.88**	**0.1**	−0.1–0.1	**1.41**	0.95–3.40	**0.3**	0.1–0.0	**0.0**	−0.3–0.2	**1.5**	0.8–2.3
Accuracy	Normal	19	**FCM**	0.8	1.5	2.2	**TAA**	0.7	1.4	2.5	**0.88**	**−0.3**	−0.9–0.1	**1.12**	0.86–1.45	**−0.1**	−0.2–0.0	**−0.1**	−0.5–0.4	**0.0**	−0.3–0.2
							**CAA**	0.8	1.5	2.2	**0.86**	**−0.1**	−0.7–0.4	**1.13**	0.69–1.60	**0.1**	−0.1–0.3	**0.0**	−0.5–0.5	**0.1**	−0.3–0.4
Accuracy	High	27	**FCM**	2.4	4.3	8.2	**TAA**	2.0	4.0	6.8	**0.91**	**0.4**	−0.5–1.1	**0.83**	0.66–1.06	**−0.3**	−0.5–0.0	**0.7**	0.1–1.4	**−0.2**	−0.4–−0.1
							**CAA**	2.2	4.1	6.9	**0.92**	**0.8**	−0.2–1.6	**0.79**	0.62–1.03	**−0.2**	−0.4–0.1	**1.0**	0.5–1.6	**−0.3**	−0.4–−0.2
Comparability	All	483	**CAA**	0.1	2.1	13.9	**TAA**	0.0	0.3	14.0	**0.98**	**−0.1**	−0.2–−0.1	**0.99**	0.97–1.01	**−0.2**	−0.2–−0.1	**−0.2**	−0.2–−0.1	**0.0**	0.0–0.0
Comparability	Low	62	**CAA**	0.1	0.4	0.9	**TAA**	0.0	0.3	0.8	**0.92**	**−0.1**	−0.1–0.0	**1.04**	0.95–1.13	**−0.1**	−0.1–0.0	**−0.1**	−0.1–0.0	**0.0**	−0.1–0.1
Comparability	Normal	273	**CAA**	0.8	1.6	2.5	**TAA**	0.7	1.4	2.8	**0.85**	**−0.2**	−0.3–−0.1	**0.99**	0.92–1.05	**−0.2**	−0.2–−0.1	**−0.2**	−0.3–−0.1	**0.0**	−0.1–0.1
Comparability	High	148	**CAA**	1.9	4.0	13.9	**TAA**	2.1	3.8	14.0	**0.97**	**−0.3**	−0.5–−0.2	**1.05**	1.01–1.09	**−0.1**	−0.2–−0.1	**−0.3**	−0.5–−0.2	**0.1**	0.0–0.1

*Note*: The percentages of reticulocytes in the clinical samples for regression analysis were classified as all, low (<0.8%), normal (0.8–2.2%), and high (>2.2%) according to the reference intervals provided by the Japanese Society for Laboratory Haematology. The assignment of samples to each group was defined by the mean value of duplicate reference values obtained by the flow cytometry reference method in the accuracy evaluation and by the mean value of measurements obtained by the comparative automated analyser and test automated analyser in the comparability evaluation. CAA, comparative automated analyser XN‐2000; FCM, flow cytometric reference method for reticulocyte count obtained by the flow cytometer FACSVia; TAA, test automated analyser Celltac G+; 95% CI, 95% confidence interval.

Abbreviations: CAA, comparative automated analyser; CI, confidence interval; FCM, flow cytometry measurement; TAA, test automated analyser.

Table 2 shows the comparability results for the TAA and CAA, and Table 3 shows the effects of potential interfering substance according to CAA flagging information and morphological observations. Regarding IP messages and NRBC in the evaluation of potential interfering substances, three samples were observed with red blood cell (RBC)‐Abn‐Distribution and eight with RET‐Abn‐Scattergram. In two samples with a high %NRBC value, that is, greater than 30, and with no IP message, the difference in %Retic was about 0.1. No outlier was present among the five samples with a high PLT count, that is, greater than 700 × 10^9^/L, or the three samples with a high WBC, that is, greater than 70 × 10^9^/L. One outlier of more than 90% between TAA and CAA was observed in %Retic. In this sample, %NRBC was high, and the TAA measurement tended to be lower than the CAA measurement. In morphological observations (Morph‐01 to 05 in Table 3), in one sample we found an outlier with a difference in %Retic of more than 110% between TAA and CAA and nucleated erythrocytes and H‐J bodies.

**TABLE 3:** Effects of interfering substances (nucleated red cells, platelets, white blood cells, and interpretive program messages) appearing in the samples in the comparative automated analyser.

	%Retic				CAA				Manual
Samples	CAA	TAA	TAA ‐CAA	(TAA–CAA)/mean	IP messages	%NRBC	PLT(×10^9^/L)	WBC (×10^9^/L)	Morphology observation
IP‐01	0.69	0.72	0.03	4%	ABN (RBC) RET Abn Scattergram	0.0	275	5.64	Not applicable
IP‐02	1.40	1.52	0.12	8%		0.0	248	3.86	
IP‐03	2.96	3.84	0.88	26%		0.3	15	7.52	
IP‐04	0.59	0.59	0.00	0%	ABN (RBC) RET AbnScattergram	0.3	22	3.82	Not applicable
IP‐05	1.27	1.28	0.01	1%		0.3	35	3.63	
IP‐06	1.74	1.56	−0.18	−11%		0.3	59	6.81	
IP‐07	2.32	1.75	−0.57	−28%		0.0	337	3.42	
IP‐08	3.40	3.36	−0.04	−1%		1.7	192	16.84	
IP‐09	6.77	2.45	−4.32	−94%		21.8	233	3.72	
IP‐10	5.11	4.99	−0.12	−2%		0.0	274	4.62	
IP‐11	9.46	8.82	−0.64	−7%		0.5	341	1.84	
NRBC‐01	0.25	0.11	−0.14	−78%	Negative	40.0	28	0.05	Not applicable
NRBC‐02	1.19	1.33	0.14	11%	Negative	32.4	37	0.74	
PLT‐01	1.26	1.06	−0.20	−17%	Negative	0.1	701	12.87	Not applicable
PLT‐02	1.16	1.39	0.23	18%	Negative	0.1	706	16.53	
PLT‐03	2.04	2.35	0.31	14%	Negative	0.1	895	31.37	
PLT‐04	1.74	1.50	−0.24	−15%	Negative	0.0	1167	14.72	
PLT‐05	3.04	2.53	−0.51	−18%	Negative	0.1	737	15.54	
WBC‐01	2.31	2.09	−0.22	−10%	Negative	0.4	469	128.22	Not applicable
WBC‐02	0.28	0.27	−0.01	−4%	Negative	0.0	50	74.84	
WBC‐03	0.33	0.23	−0.10	−36%	Negative	0.0	52	74.20	
Morph‐01	7.97	2.20	−5.77	−113%	ABN (RBC) RET Abn Scattergram	‐‐‐	312	5.14	Nucleated erythrocytes, Howell‐Jolly bodies
Morph‐02	1.60	1.18	−0.42	−30%	Negative	‐‐‐	181	2.44	Howell‐Jolly bodies
Morph‐03	1.43	1.24	−0.19	−14%	ABN (WBC) NRBC Present	28.7	20	1.22	Nucleated erythrocytes
Morph‐04	4.71	4.02	−0.69	−16%	Negative	0.2	201	6.21	Fragmented erythrocytes
Morph‐05	2.28	0.82	−1.46	−94%	ABN (PLT) PLT DistributionABN (WBC) NRBC Present	4.0	40	0.75	Giant platelets

Abbreviations: CAA, comparative automated analyser XN‐2000; %NRBC, percentage of nucleated red blood cells; %Retic, percentage of reticulocytes; PLT, platelet count; TAA, test automated analyser Celltac G+; WBC, white blood cell count.

## 4 DISCUSSION

The study validated the basic performance of the AO metachromatic staining‐based TAA with RNP for measuring reticulocytes.

In the preliminary evaluation, which focused on the low range of %Retic, the correlation criteria for AMI and accuracy were satisfied. In addition, when the *SD* of %Retic was 0.01, the LoQ of the TAA was 0.14. Hence, the minimum detection sensitivity of the TAA was sufficient for use in patients with acute normocytic anaemia due to acute parvovirus B19 infection.

Immature reticulocytes reflect erythrocytes in the early phase of hematopoietic recovery after hematopoietic stem cell transplantation.^20^ Immature reticulocyte fraction (IRF) was suggested for monitoring immature reticulocytes, but the calculated value still has not been standardized. Consequently, reticulocyte count typically is used as an alternative to IRF.

The ability to measure low reticulocytes levels is clinically important. In the comprehensive evaluation of the present study, the correlation criteria for AMI and accuracy were satisfied for all %Retic ranges, and no differences were observed in the overall, low, or normal groups between the TAA and FCM. Thus, this evaluation showed good performance of the TAA, similar to the performance found in the low %Retic range in the preliminary evaluation. Regarding the upper limit required for the AMI of %Retic, a guideline publication described how reticulocytes increase in response to anaemia: %Retic is 5.0 or higher if the reticulocyte level is 3.00 × 10^12^/L or higher, assuming the RBC count is 2.00 × 10^12^/L^2^. In autoimmune hemolytic anaemia, which shows a greater increase in %Retic, a study reported that the median %Retic at diagnosis in 109 cases was 9%.^21^, ^22^ Hence, in the present study the verified AMI of the TAA (%Retic: 0.03 to 8.23) mostly covers the expected clinical range of values. In the high %Retic groups, measurements obtained with both the TAA and the CAA appeared to be lower than those obtained with FCM, even though the two HAs use different mechanisms. The cause of this negative bias was not identified because of the small number of clinical specimens obtained within the evaluation period. Future studies need to include more specimens, for example, by evaluating enriched blood from healthy specimens.

In the comparability evaluation, correlation criteria were satisfied in the negative group without a large deviation, in the overall group, and in all three concentration groups. However, some deviations were observed in the positive samples. In one case, a large deviation was observed: %Retic was markedly higher with the CAA (6.77) than with the TAA (2.45), and an IP message (Ret‐Abn‐Scattergram) and high %NRBC (21.8%) were observed with the CAA.

In morphology assessment, a similar case with large deviation was observednucleated erythroblasts (17/100 WBC) and H‐J bodies (20%) were seen. In this case, %Retic was higher with CAA (7.97) than with TAA (2.20). In four typical cases with a small deviation between the TAA and CAA measurements, %Retic tended to be higher with CAA than with TAA, and potential interfering substances (erythroblasts, H‐J bodies, fragmented erythrocytes, and high platelet count) were observed also in these cases.

In the two samples with a large deviation, when both fluorescence histograms of erythrocyte events in the TAA were compared with the distribution of normal samples, no abnormal distribution was observed on the red fluorescence histogram (which reflects immaturity); however, on the green fluorescence histogram both samples showed a distribution of DNA‐containing cells that indicated abnormal erythrocyte morphology. These findings suggest that erythrocyte morphological abnormality can be detected on the basis of the fluorescence ratio. The RNA and DNA staining on the TAA likely reflects the components of the reticulocyte staining seen in typical HA's; that is, it might be expressing the information of the immaturity and the amount of DNA contents in cells.

Regarding the effects of potential interfering substances, in 6 of 8 samples that triggered the IP message Ret‐Abn‐Scattergram, %Retic was lower with the TAA than with the CAA, and the deviation was associated with a difference in %NRBC count. In addition, some deviations were observed in two samples without IP messages but with a %NRBC count of 30% or higher: In NRBC‐01, many deviations occurred, although they were small; in contrast, in NRBC‐02, no large deviations in %Retic were observed in the normal range. For samples with high platelet counts or high WBC counts, no large deviations were observed between TAA and CAA, indicating that these characteristics have little effect on measurements of reticulocytes. The deviation ratios of %Retic appeared to be higher with TAA than with CAA, particularly in the low range. The LoQ of the TAA, CAA, and FCM described sufficient minimum detection sensitivity. In addition, the mean %Retic in the three samples with a %Retic of less than 0.1 were 0.03 with FCM, 0.05 with TAA, and 0.15 with CAA. Taken together, these results indicate that the deviation might reflect the effect of spurious reticulocyte counts.

In this study, measurements of reticulocytes by Celltac G+ demonstrated good correlation with the reference method (FCM) and routine HA (CAA). The deviations in the values measured by the two HAs may be due to DNA‐containing erythrocytes in abnormal samples and is likely related to the difference in the measurement method. Therefore, before using Celltac G+ in clinical practice, we need to fully understand the measurement method and how it may affect the interpretation of results.

## 5 CONCLUSION

Celltac G+, a novel HA that determines RNP with metachromatic nucleic acid staining with AO, uses a different measurement approach than conventional analyzers and can measure reticulocytes more accurately. Regarding its use for clinical testing, Celltac G+ is expected to be introduced as a new HA and to provide novel, useful messages on immaturity and morphological abnormality of erythrocytes.

## AUTHOR CONTRIBUTIONS

Kaori Yahagi and Tomoko Arai equally contributed to this work, and both should be considered first authors. Yutaka Nagai, Hisako Katagiri conceived the study. Kaori Yahagi, Tomoko Arai, Hisako Katagiri and Yutaka Nagai designed the study. Kaori Yahagi, Tomoko Arai, Hisako Katagiri, Yoko Yatabe and Yutaka Nagai performed the experiments. Kaori Yahagi, Tomoko Arai, Hisako Katagiri and Yutaka Nagai analysed and interpreted the data. Kaori Yahagi, Tomoko Arai, Hisako Katagiri, and Yutaka Nagai wrote the manuscript. Kaori Yahagi, Tomoko Arai, Hisako Katagiri, Yutaka Nagai, Yoko Yatabe, Hiromitsu Yokota, Takayuki Mitsuhashi, Masatoshi Wakui and Mitsuru Murata discussed the data and critically reviewed and revised the manuscript. Kaori Yahagi, Tomoko Arai, Hisako Katagiri and Yutaka Nagai wrote the paper. Yutaka Nagai researched literature for review and wrote the initial paper. All authors declare that they had full access to the data to revise and approve the manuscript.

## CONFLICT OF INTEREST

This work was supported by Nihon Kohden corporation. Yutaka Nagai was employee of Nihon Kohden corporation.

## Supporting information


Appendix A1 Repeatability

**Appendix A2** Precision profile in the analytical measurement interval

## Data Availability

The data that support the findings of this study are available from the corresponding author upon reasonable request.
