# Supplementary material for: Performance evaluation of a novel reticulocyte identification method that uses metachromatic nucleic acid staining based on a crossover analysis of emission DNA/RNA light (RNP Determination™) in hematology analyzer Celltac G+
Source: Int J Lab Hematol. 2022 Aug 17;44(6):1050–9. doi: 10.1111/ijlh.13947 (PMC9804789; doi:10.1111/ijlh.13947)
Supplement: Supplementary file 1 — Appendix A1 Repeatability [file IJLH-44-1050-s002.pptx]

## Slide 1
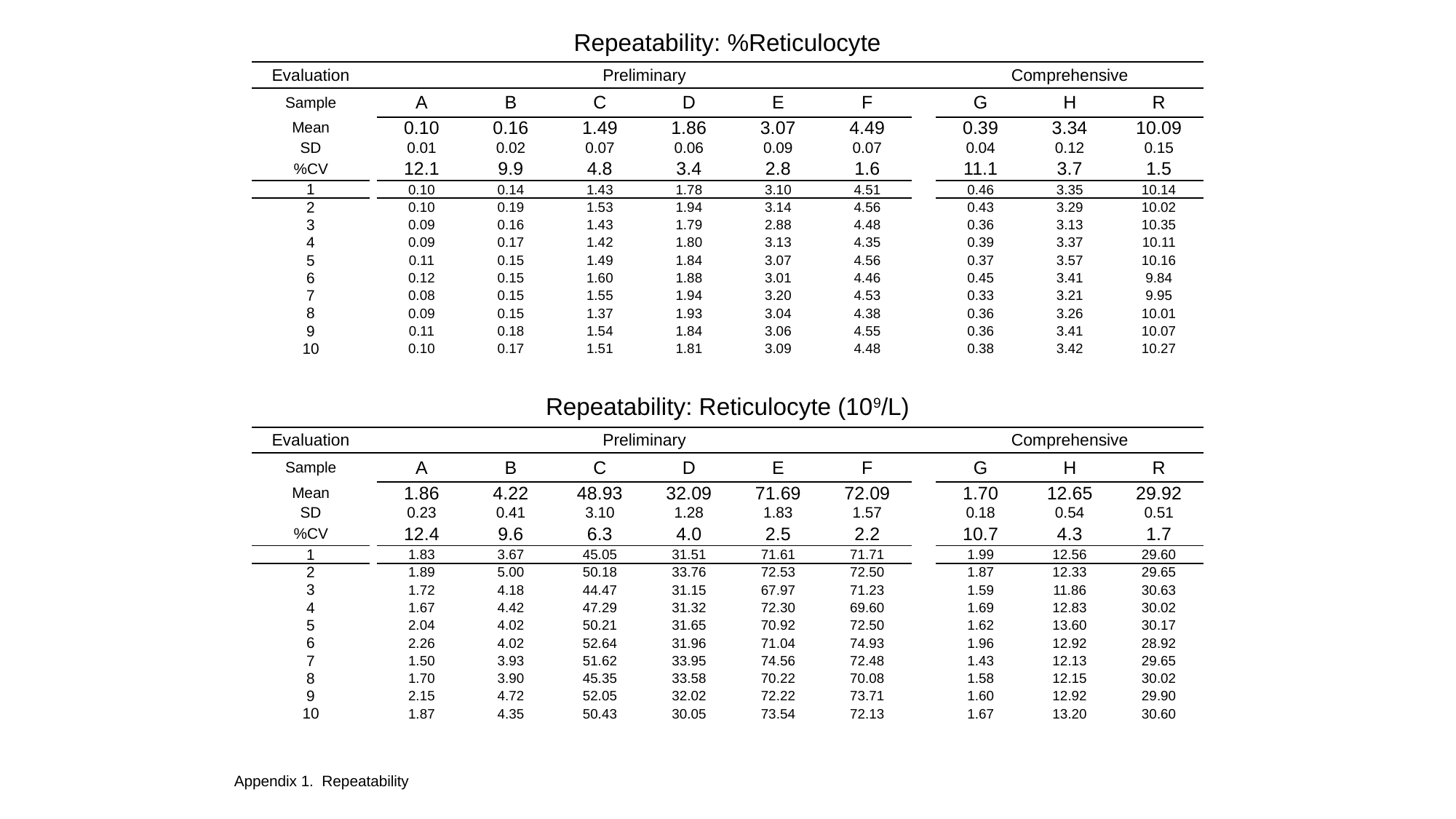

| Repeatability: %Reticulocyte | | | | | | | | | | | |
| --- | --- | --- | --- | --- | --- | --- | --- | --- | --- | --- | --- |
| Evaluation | | Preliminary | | | | | | | Comprehensive | | |
| Sample | | A | B | C | D | E | F | | G | H | R |
| Mean | | 0.10 | 0.16 | 1.49 | 1.86 | 3.07 | 4.49 | | 0.39 | 3.34 | 10.09 |
| SD | | 0.01 | 0.02 | 0.07 | 0.06 | 0.09 | 0.07 | | 0.04 | 0.12 | 0.15 |
| %CV | | 12.1 | 9.9 | 4.8 | 3.4 | 2.8 | 1.6 | | 11.1 | 3.7 | 1.5 |
| 1 | | 0.10 | 0.14 | 1.43 | 1.78 | 3.10 | 4.51 | | 0.46 | 3.35 | 10.14 |
| 2 | | 0.10 | 0.19 | 1.53 | 1.94 | 3.14 | 4.56 | | 0.43 | 3.29 | 10.02 |
| 3 | | 0.09 | 0.16 | 1.43 | 1.79 | 2.88 | 4.48 | | 0.36 | 3.13 | 10.35 |
| 4 | | 0.09 | 0.17 | 1.42 | 1.80 | 3.13 | 4.35 | | 0.39 | 3.37 | 10.11 |
| 5 | | 0.11 | 0.15 | 1.49 | 1.84 | 3.07 | 4.56 | | 0.37 | 3.57 | 10.16 |
| 6 | | 0.12 | 0.15 | 1.60 | 1.88 | 3.01 | 4.46 | | 0.45 | 3.41 | 9.84 |
| 7 | | 0.08 | 0.15 | 1.55 | 1.94 | 3.20 | 4.53 | | 0.33 | 3.21 | 9.95 |
| 8 | | 0.09 | 0.15 | 1.37 | 1.93 | 3.04 | 4.38 | | 0.36 | 3.26 | 10.01 |
| 9 | | 0.11 | 0.18 | 1.54 | 1.84 | 3.06 | 4.55 | | 0.36 | 3.41 | 10.07 |
| 10 | | 0.10 | 0.17 | 1.51 | 1.81 | 3.09 | 4.48 | | 0.38 | 3.42 | 10.27 |
| | | | | | | | | | | | |
| Repeatability: Reticulocyte (109/L) | | | | | | | | | | | |
| Evaluation | | Preliminary | | | | | | | Comprehensive | | |
| Sample | | A | B | C | D | E | F | | G | H | R |
| Mean | | 1.86 | 4.22 | 48.93 | 32.09 | 71.69 | 72.09 | | 1.70 | 12.65 | 29.92 |
| SD | | 0.23 | 0.41 | 3.10 | 1.28 | 1.83 | 1.57 | | 0.18 | 0.54 | 0.51 |
| %CV | | 12.4 | 9.6 | 6.3 | 4.0 | 2.5 | 2.2 | | 10.7 | 4.3 | 1.7 |
| 1 | | 1.83 | 3.67 | 45.05 | 31.51 | 71.61 | 71.71 | | 1.99 | 12.56 | 29.60 |
| 2 | | 1.89 | 5.00 | 50.18 | 33.76 | 72.53 | 72.50 | | 1.87 | 12.33 | 29.65 |
| 3 | | 1.72 | 4.18 | 44.47 | 31.15 | 67.97 | 71.23 | | 1.59 | 11.86 | 30.63 |
| 4 | | 1.67 | 4.42 | 47.29 | 31.32 | 72.30 | 69.60 | | 1.69 | 12.83 | 30.02 |
| 5 | | 2.04 | 4.02 | 50.21 | 31.65 | 70.92 | 72.50 | | 1.62 | 13.60 | 30.17 |
| 6 | | 2.26 | 4.02 | 52.64 | 31.96 | 71.04 | 74.93 | | 1.96 | 12.92 | 28.92 |
| 7 | | 1.50 | 3.93 | 51.62 | 33.95 | 74.56 | 72.48 | | 1.43 | 12.13 | 29.65 |
| 8 | | 1.70 | 3.90 | 45.35 | 33.58 | 70.22 | 70.08 | | 1.58 | 12.15 | 30.02 |
| 9 | | 2.15 | 4.72 | 52.05 | 32.02 | 72.22 | 73.71 | | 1.60 | 12.92 | 29.90 |
| 10 | | 1.87 | 4.35 | 50.43 | 30.05 | 73.54 | 72.13 | | 1.67 | 13.20 | 30.60 |
Appendix 1. Repeatability
