# Supplementary material for: Performance evaluation of a novel reticulocyte identification method that uses metachromatic nucleic acid staining based on a crossover analysis of emission DNA/RNA light (RNP Determination™) in hematology analyzer Celltac G+
Source: Int J Lab Hematol. 2022 Aug 17;44(6):1050–9. doi: 10.1111/ijlh.13947 (PMC9804789; doi:10.1111/ijlh.13947)
Supplement: Supplementary file 2 — Appendix A2 Precision profile in the analytical measurement interval [file IJLH-44-1050-s001.pdf]

Sample	n	Mean	SD	95% CI	CV
LOQ1	10	0.099	0.012	0.008 to 0.022	12.1%
LOQ2	10	0.161	0.016	0.011 to 0.029	9.9%
LOQ3	10	1.487	0.072	0.049 to 0.131	4.8%
LOQ4	10	1.855	0.063	0.044 to 0.116	3.4%
LOQ5	10	3.072	0.087	0.060 to 0.158	2.8%
LOQ6	10	4.486	0.073	0.050 to 0.133	1.6%

Model	2-parameter linear variance function	
Equation	σ^2 = ß0 + ß1	
	β_0= -9.148 E-05	
	β_1= 0.002324	
-LogLikelihood	2.6482	
Inverse Prediction	CV	11.0%
	LoQ	0.14

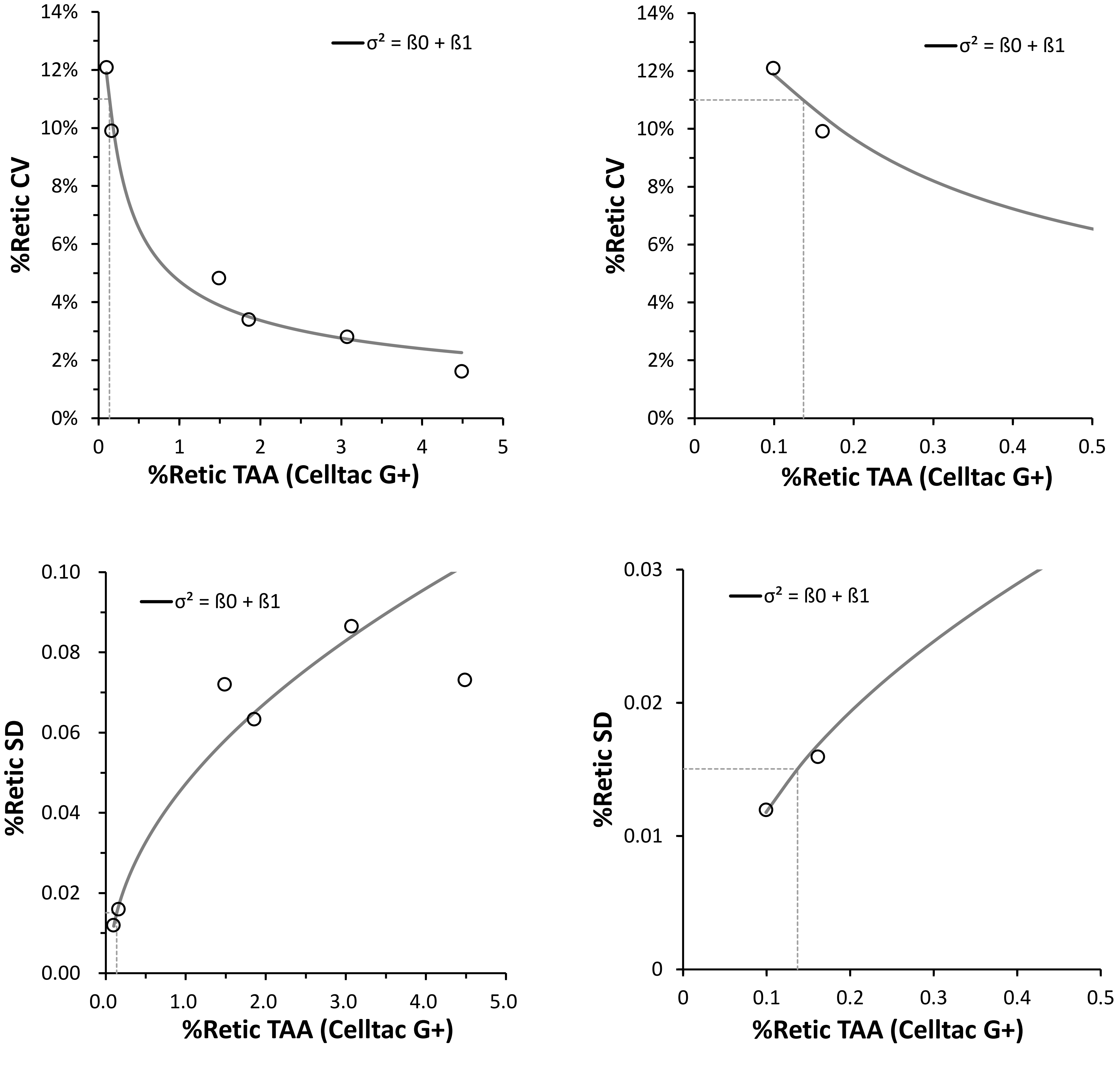

Appendix 2 Precision profile in the analytical measurement interval
The limit of quantity in the percentage of reticulocytes as the coefficient of variation within the 11% limit (SD within 0.01 limit) was defined as 0.14.
